# The semi-sitting position in patients with indwelling CSF shunts: perioperative management and avoidance of complications

**DOI:** 10.1007/s00701-022-05430-4

**Published:** 2022-12-11

**Authors:** Manolis Polemikos, Paul Bronzlik, Hans E. Heissler, Elvis J. Hermann, Shadi Al-Afif, Majid Esmaeilzadeh, Joachim K. Krauss

**Affiliations:** 1 Department of Neurosurgery, Hannover Medical School, Carl-Neuberg-Str. 1, DE-30625 Hannover, Germany; 2 Institute for Diagnostic and Interventional Neuroradiology, Hannover Medical School, Hannover, Germany

**Keywords:** Hydrocephalus, Semi-sitting position, Ventriculoperitoneal, Shunt, Pneumocephalus

## Abstract

**Objective:**

Posterior fossa or midline tumors are often associated with hydrocephalus and primary tumor removal with or without perioperative placement of an external ventricular drain (EVD) is commonly accepted as first-line treatment. Shunting prior to posterior fossa surgery (PFS) is mostly reserved for symptomatic cases or special circumstances. There are limited data regarding the anticipated risk for symptomatic pneumocephalus and the perioperative management using the semi-sitting position (SSP) in such a scenario. Here, we therefore assessed the safety of performing PFS in a consecutive series of patients over a period of 15 years to allow the elaboration of recommendations for perioperative management.

**Methods:**

According to specific inclusion and exclusion criteria a total of 13 patients who underwent 17 operations was identified. Supratentorial pneumocephalus was evaluated with semiautomatic-volumetric segmentation. The volume of pneumocephalus was evaluated according to age and ventricular size.

**Results:**

Ten of the 13 patients had a programmable valve (preoperative valve setting range 6–14 cmH20; mean 7.5 cmH20) while 3 patients had non programmable valves. A variable amount of supratentorial air collection was evident in all patients postoperatively (range 3.2–331 ml; mean 122.32 ml). Positive predictors for the volume of postoperative pneumocephalus were higher age and a preoperative Evans ratio > 0.3. In our series, we encountered no cases of tension pneumocephalus necessitating an air replacement procedure as well as no obstruction, disconnection, infection or hardware malfunction of the shunt system.

**Conclusions:**

Our findings indicate that a CSF shunt in situ is not a contraindication for performing PFS in the semi-sitting position and it does not increase the pre-existing risk for postoperative tension pneumocephalus. In cases of primary shunting for hydrocephalus associated with posterior fossa tumors a programmable valve set at a medium opening pressure with a gravitational device is a valid option when PFS in the semi-sitting position is opted. In patients with an indwelling shunt diversion system special caution is indicated in order to prevent and detect overdrainage especially in not adjustable valves or shunts without antisiphon devices.

## Introduction

Posterior fossa tumors are often associated with hydrocephalus at presentation, which commonly resolves after tumor removal. Nevertheless, cerebrospinal fluid (CSF) shunting may be mandatory prior to tumor resection in symptomatic cases [2, 4, 27]. Furthermore, hydrocephalus may be attributable to concomitant hydrocephalus not related to the posterior fossa tumor in the rare case, necessitating permanent CSF diversion.

While the semi-sitting position (SSP) is one of the preferred positions when performing posterior fossa surgery (PFS), it may be associated with postoperative symptomatic (tension) pneumocephalus [6, 13, 15, 18, 26].

This raises the question whether a CSF shunt in situ would increase the imminent risk of postoperative symptomatic pneumocephalus or if it would be associated with additional complications.

Thus far, this problem has achieved relatively little attention [22], and it has even been suggested to avoid operating on patients with CSF shunts in the SSP [8, 20]. Here, we aimed to assess the safety of the SSP when performing PFS in previously shunted patients over a period of 15 years. In addition, based on our findings, recommendations for perioperative management of these patients are proposed.

## Methods

We analyzed a prospectively maintained electronic database of patients with posterior fossa lesions who were operated in the SSP between 2005 and 2020 using a retrospective study design. Inclusion criteria consisted of the following: 1. patients with a (functioning) in situ CSF shunt placed > 1 week prior to PFS in the semi-sitting position, 2. age > 18 years at the time of PFS, and 3. available preoperative and postoperative neuroimaging. Patients operated in the SSP after endoscopic third ventriculostomy or with an indwelling external ventricular drain (EVD) were excluded in view of the fact that the EVD was routinely clamped prior to patient positioning. Cervical spine operations or deep brain stimulation procedures were also not included in this study.

For each patient, medical and radiological records were reviewed for the following information: patient demographics; type of CSF diversion (ventriculoperitoneal (VP) shunt or ventriculoatrial (VA) shunt); valve-type, valve setting, and perioperative valve and shuntmanagement; Evans ratio at the time of PFS; surgical data (posterior fossa lesion pathology, surgical approach, operating time) and postoperative course.

Postoperative pneumocephalus was determined with semiautomatic volumetric segmentation on Visage® 7 (Visage Imaging Inc.) on a head CT performed according to a standard protocol 6 to 12 h after PFS.

Patient consent was not required for this retrospective study with no identifiable patient data. All operations were performed by senior neurosurgeons of our department specialized in posterior fossa surgery according to standard operative procedures as outlined elsewhere [1]. The SSP was chosen with regard to tumor location, the option for a more advantageous access, and clinical judgment.

In order to determine predictors for the volume of postoperative pneumocephalus a statistical comparison of continuous variables was conducted using Student’s *t*-test (two-sided). A *p*-value of 0.05 was set as threshold for statistical significance.

## Results

### Patient characteristics

Over a period of 15 years a total of 13 patients (10 women and 3 men) with a CSF shunt in situ placed prior to PFS were operated overall 17 times in the SSP. The mean patient age at PFS was 46.7 years, ranging from 23 to 70 years. Patient characteristics and clinical data including hydrocephalus diagnoses are shown in Tables 1 and 2.

**Table 1:** Individual surgeries performed in the semi-sitting position in patients with a CSF shunt

PFS Nr	Age/sex	Posterior fossa pathology	Approach	OP time (min)	Months after shunt surgery	Shunt type/valve	ER	PC (ml)
1	56/f	VS/T4b	LSO	393	0.5	VP/proGAV 2.0 + gravitational unit	0.407	132.2
2	68/f	VS/T4a	LSO	242	1	VP/Medtronic delta 1.0	0.386	250.2
3	45/f	VS/T4a	LSO	210	51	VP/proGAV + gravitational unit	0.407	331
4	32/f	VS/T4a/RS/NFII/recurrent	LSO	427	73	VP/proGAV + gravitational unit	0.274	20.7
5	56/m	VS/T3b	LSO	315	0.5	VP/proGAV + gravitational unit	0.409	123.3
6	60/f	VS/T4a	LSO	250	3	VP/Codman Hakim adjustable	n.a	n.a
7a	36/f	PTPR	MSO	180	119	VA/Codman-Hakim- medium pressure	n.a	n.a
7b	37/f	PTPR	MSO	160	136	VA/Codman-Hakim-medium pressure	0.253	41.2
7c	39/f	PTPR	MSO	250	154	VA/Codman-Hakim-medium pressure	0.269	35.6
7d	40/f	PTPR	MSO	230	162	VA/Codman-Hakim-medium pressure	0.277	139.9
8	44/f	Metastasis (tectum)	MSO	407	12	VP (parietal)/not identified	0.247	118.8
9	70/m	Meningioma (falcotentorial)	MSO	405	0.75	VP/Codman Certas Plus	0.282	118.7
10	65/f	Meningioma (petroclival)	LSO	415	5	VP/proGAV + gravitational unit	0.363	170.6
11	47/m	Hemangioblastoma(midline)	MSO	185	72	VP/proGAV + gravitational unit	0.249	75.4
12a	23/f	Ganglioglioma (tectum)	MSO	265	150	VP/Codman-Hakim adjustable	0.296	77.4
12b	23/f	Ganglioglioma (tectum)	MSO	325	155	VP/ Codman-Hakim adjustable	0.316	196.6
13	55/f	Ruptured aneurysm (PICA)	MSO	113	19	VP/Codman-Hakim adjustable	0.233	3.2

*ER* Evans ratio, *F* female, *LSO* lateral suboccipital, *M* male, *MSO* median suboccipital, *NF* neurofibromatosis, *PC* pneumocephalus, *PICA* posterior inferior cerebellar artery, *PTPR* papillary tumor of pineal region, *RS* radiosurgery, *VA* ventriculoatrial, *VP* ventriculoperitoneal, *VS* vestibular schwannoma

**Table 2:** Overview of patients with a CSF shunt operated in the semi-sitting position

Variable	Value
Age at surgery in years (range)	45.9 (23–70)
Sex (Male:Female)	3:10
Mean postoperative pneumocephalus volume (ml)	122.32 (3.2–331)
Mean surgical procedure time in min (range)	250 (113–427)
Shunt characteristics/total PFS performed	
Adjustable with fixed gravitational unit	6/6
Adjustable w/o gravitational unit	4/5
Not adjustable (medium pressure)	1/4
Siphon control valve	1/1
Not identified	1/1
Posterior fossa lesions/total PFS performed	
Vestibular schwannoma (T3b = 1;T4a = 4;T4b = 1)	6/6
Meningioma (petroclival = 1; falcotentorial = 1)	2/2
Papillary Tumor of the pineal region (PTRT)	1/4
Hemangioblastoma (brainstem, recurrent)	1/1
Ganglioglioma (tectum/midbrain)	1/2
Metastasis (tectum/velum interpositum)	1/1
Aneurysm (ruptured) (posterior inferior cerebellar artery)	1/1

### CSF diversion

CSF shunts had been placed for communicating hydrocephalus in seven and for obstructive hydrocephalus in four patients, respectively, whereas a combined occlusive/malabsorptive pathophysiology was evident in two instances. The time between shunt surgery and PFS ranged from 2 weeks to 162 months (mean: 65.6 months). A VP shunt had been installed in 12/13 patients, inserted via a precoronal burr hole in 11/12 cases, and through a parietal burr hole in one instance. One patient with a papillary tumor of the pineal region (PTPR) underwent a total of 4 PFS for tumor resection in the SSP over the course of 14 years after initial placement of a VA shunt [14]. Hydrocephalus was attributable to the underlying posterior fossa pathology in all but 3 instances. These included two patients with communicating hydrocephalus secondary to an aneurysmal subarachnoid hemorrhage 5 and 2 years prior PFS in the SSP and one patient with post-meningitis hydrocephalus which had occurred after PFS for tumor resection 6 years earlier. In these patients PFS was performed for resection of a vestibular schwannoma and for clipping of a ruptured aneurysm, respectively.

### Valve type and preoperative shunt management

For all cases, except one, information on the manufacturer and the type of valve was available. One international patient had a fixed pressure valve, which was lacking documentation and could not be identified radiologically.

Ten patients had a programmable differential pressure valve, including 6 with an integrated fixed gravitational device (ProGAV, Miethke-Aesculap, Germany). The preoperative valve settings ranged between 6 and 14 cm H20 (mean 7.5 cm H20). Further shunt types included a fixed medium pressure valve and a siphon control valve, in one instance respectively.

### Surgery

Patients were positioned in the SSP with the head fixed in a three-pin Mayfield clamp and the legs elevated at heart level while the knees were slightly flexed. During head fixation caution was given to avoid injury of the underlying shunt. Standard intraoperative multimodal monitoring included somatosensory evoked potentials (SSEP), electromyography (EMG), and brainstem auditory evoked potentials (BAEP) dependent on the localization of the lesion as previously described [13]. PFS was performed by standard microsurgical procedures [1]. A median/paramedian suboccipital craniotomy was performed in 10 instances, whereas the retrosigmoid approach was utilized 7 times for removal of 6 vestibular schwannomas and one petroclival meningioma. PFS was performed for an extraaxial posterior fossa lesion in 10 patients, whereas 3 patients had an intraaxial lesion resulting in a total of 13 and 4 operations respectively in the semi-sitting position. PFS involved removal of a tumor in all but one vascular case, in whom microsurgical clipping of a ruptured aneurysm of the posterior inferior cerebellar artery was performed.

### Preoperative and perioperative shunt management

Shunt management prior to PFS was based on the clinical findings of each patient rather than the size of the ventricles. In patients with adjustable shunt valves the opening pressure was not altered preoperatively. The Evans ratio prior to PFS ranged from 0.233 to 0.409 (median 0.288).

One patient with obstructive hydrocephalus due to a falcotentorial meningioma presented in our department after implantation of a VP shunt in another clinic. Preoperative imaging revealed overdrainage with bifrontal asymptomatic subdural hygromas. After gross total resection via a combined infratentorial/supracerebellar approach with an occipital transtentorial extension the shunt was ligated behind the ear to prevent further overdrainage but also to allow postoperative rapid reconnection in case of persisting shunt dependency.

### Immediate postoperative findings

After PFS, patients were transferred from the operation theater to the neurosurgical intensive care unit. Anesthesia was thereafter discontinued in all except in one patient with aneurysmal subarachnoid hemorrhage Hunt and Hess Grade 5. After extubation patients were monitored for at least one night. The median stay in the ICU was 2.5 days. CT scans performed within 6–12 h after PFS ruled out postoperative or shunt-related complications in all patients.

### Pneumocephalus

**Fig. 1:**
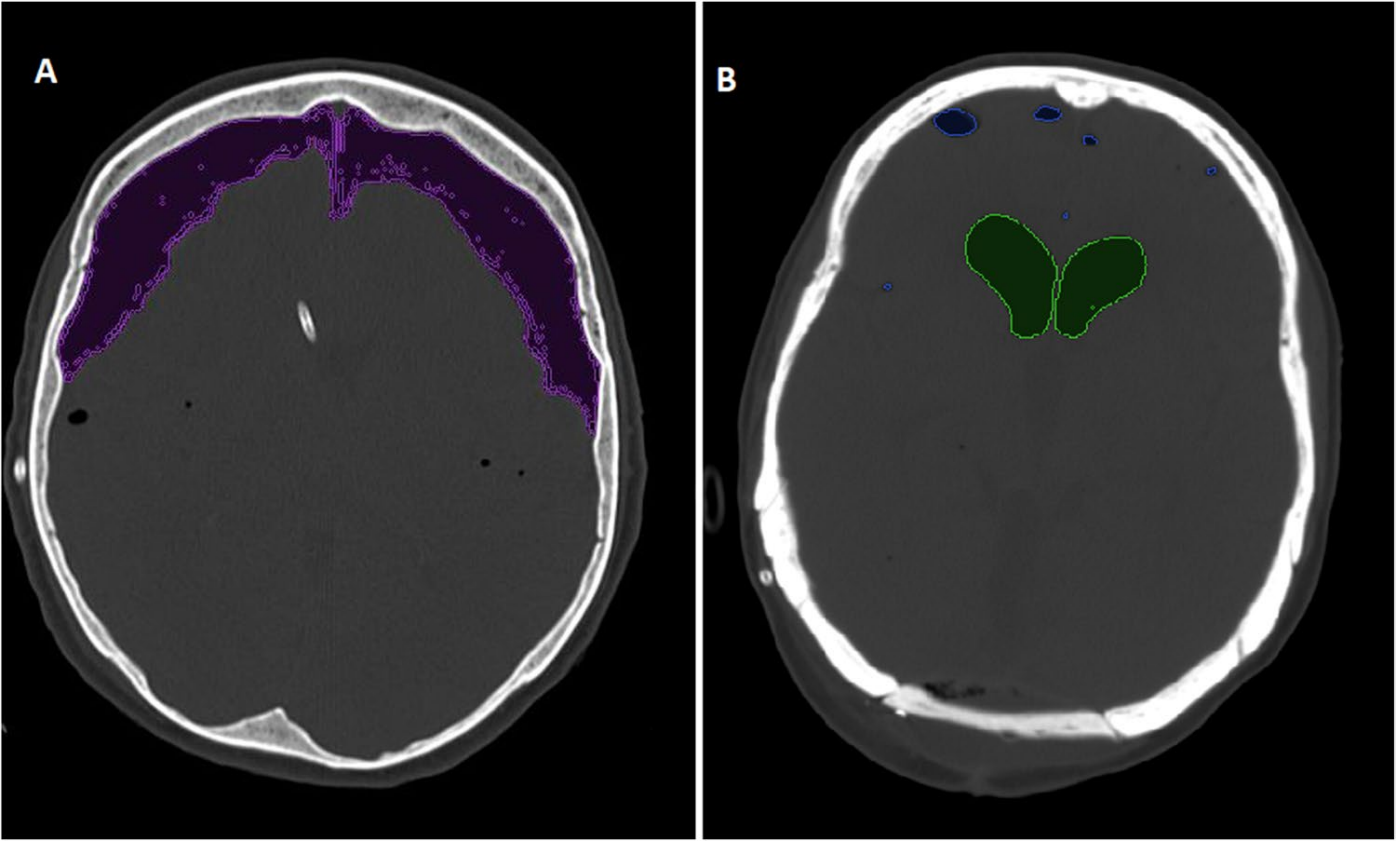
Segmentation of postoperative pneumocephalus demonstrating subdural (**A**) and intraventricular air collection (**B**)

Isolated supratentorial subdural or ventricular air entrapment was evident after 8 and 2 PFS, respectively, while in 7 instances subdural and ventricular air collections were encountered. The exact amount of postoperative pneumocephalus was determined via semiautomatic volumetric segmentation in all but 2 early cases, in which the postoperative CT scan could not be retrieved in digital form (Fig. 1). The mean amount of supratentorial postoperative intracranial air was 122.32 ml (range 3.2–331 ml).

A variable amount of supratentorial air collection was evident in all patients (Table 1). None of the patients, however, developed tension pneumocephalus which necessitated air replacement surgery via an external ventricular drain. Patients received postoperative ventilation with 100% oxygen when deemed necessary [13].

### Early postoperative findings

Early (< 30 days) postoperative clinical or radiological signs of overdrainage were evident in 2 patients after resolution of postoperative pneumocephalus and resulted in the formation of subdural hygromas. In one instance, successive adjustment of the valve opening pressure from 8 to 16 cm H20 resulted in symptom relief and resolution of subdural hygromas. In the other patient with a non-adjustable valve postoperative overdrainage with subdural hygromas was noted 2 weeks after PFS. Thereafter the shunt was ligated, however, when signs of shunt dependency became manifest, the non-programmable valve was replaced by an adjustable valve and an integrated gravitational device.

### Late postoperative findings

During a mean follow-up period of 43.2 months (range 2–108) one patient showed late (> 30 days after PFS) symptoms of overdrainage. In this patient the valve opening pressure was increased from 14 to 20 cm H2O. In the patient with the ligated VP shunt after falcotentorial meningioma surgery, ventricular size had further decreased at 1-year follow-up, and the shunt system was explanted.

### Predictors for the volume of postoperative pneumocephalus

One patient with a postoperative pneumocephalus of more than 300 ml was classified as an outlier and was not included in the comparative statistics.

Patients older than 60 years demonstrated significantly higher (p = 0.021) total pneumocephalus volumes (mean = 162.7 ± 67.2 ml) than patients younger than 60 years (mean = 76.7 ± 53 ml).

**Fig. 2:**
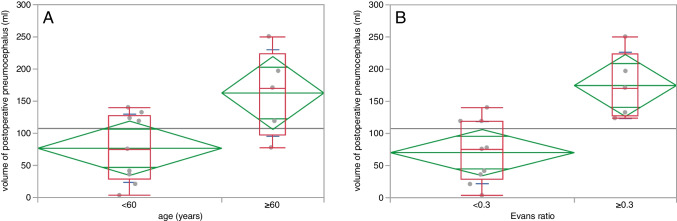
Box plots of the mean postoperative pneumocephalus volume demonstrating a significantly higher volume of postoperative pneumocephalus in patients older than 60 years (**A**) and in patients with a preoperative Evans ratio ≥ 0.3 (**B**)

Significantly higher (*p* = 0.026) pneumocephalus volumes were also seen in patients with an Evans ratio > 0.3 (mean = 174.6 ± 51.6 ml, vs mean = 70.1 ± 48.3 ml) (Fig. 2)

## Discussion

In the present study, we concentrate primarily on the findings relevant to the presence of a shunt system when performing PFS in the SSP but not on the controversial issues of optimal management of hydrocephalus associated with posterior fossa lesions or the ideal patient positioning for PFS [18]. These subjects are a matter of ongoing debate and therefore left out of the main scope of this article [1].

The frequency of hydrocephalus associated with PFT varies depending on patients’ age and tumor entity. In adult patients hydrocephalus associated with PFT can be evident in up to 21.4% and commonly resolves postoperatively. Nevertheless, preoperative treatment remains mandatory in symptomatic patients. Treatment options include perioperative placement of an external ventricular drain, endoscopic third ventriculostomy or the insertion of a CSF shunt [2, 4, 19].

Pneumocephalus after PFS is mainly attributable to the “inverted pop bottle” mechanism in combination with an increased CSF loss [16]. In addition, patients’ age, duration of surgery, male gender, nitrous oxide (N_2_O) anesthesia and continuous CSF drainage via a lumbar drain are known risk factors [13, 18, 21]. Although pneumocephalus is common when surgery is performed in the SSP it is rarely symptomatic. In general, the incidence of tension pneumocephalus after PFS in the SSP is low (0–3.3%) and not dependent on the exact amount of intracranial air [5, 12, 18, 22, 24]. These findings are in concordance with our results. Although in our study postoperative supratentorial pneumocephalus occurred in all patients, patients remained neurologically intact even when extensive intracranial air volumes were encountered. Nevertheless, in those patients ICU stay was prolonged precautionary in order to ensure close neurological monitoring.

Besides patient positioning, an indwelling functioning CSF shunt has been considered a contributing factor for the development of pneumocephalus [3, 5, 22, 25]. Previous reports have indicated that PFS in SSP is hazardous and should be avoided in previously shunted patients [8, 20]. Thus, the anticipated risk of postoperative symptomatic pneumocephalus or the occurrence of additional complications (i.e., intracranial hematomas) in patients with an indwelling CSF shunt has not been sufficiently studied before. In addition, previous reports did not address both quantitative and qualitative data such as the type of implanted valves or the use of anti-siphon devices. Furthermore, no centre has yet provided a large enough series from which to draw more definitive conclusions regarding the perioperative management of this particular group of patients.

Remarkably, Sloan et al. found that the presence of a VP shunt or an external ventricular drain did not increase the volume of supratentorial pneumocephalus when measured within 4 h, although in 4 patients extensive supratentorial air was evident when pneumocephalus was measured at one day postoperatively or later [22].

In our study the most common (6/13) pathology operated was a vestibular schwannoma, corresponding to higher tumor extension grades T3b, T4a, and T4b in one, four, and one case respectively. With that regard, it needs to be mentioned, that Machetanz noted T4 tumors to be a negative predictor of postoperative pneumocephalus after removal in the SSP [18]

Our experience in performing PFS in the SSP [1, 9, 11, 13] as well as our present observations allows us to make the following recommendations:

First, if a VP shunt is indicated prior to PFS in the SSP, an adjustable valve with a fixed gravitational unit is preferable since such a combination has been proven to reduce overdrainage especially upon postural changes [7, 10, 23]. Depending on the severity of clinical symptoms a medium opening pressure setting appears to be a valid option. Further preoperative adjustments of the shunt valve can be performed when clinical or radiological signs of under- or overdrainage are evident. Shunted patients with an preoperative Evans ratio > 0,3 are susceptible for higher postoperative intracranial air volumes. In patients with an indwelling non-adjustable shunt, valve replacement with a programmable valve and an antisiphon device may be considered when hydrocephalus is unlikely to resolve after PFS or overdrainage is anticipated due to postsurgical altered CSF dynamics.

Postoperative CT is advisable in all patients to detect acute complications. In patients with reduced arousal after discontinuation of anesthesia special caution is needed in order to detect overdrainage or tension pneumocephalus. Endotracheal administration of normobaric hyperoxia for 3 h in the early postoperative phase has been proven safe and efficacious as primary treatment of pneumocephalus after PFS, and it can also be applied in previously shunted patients with pneumocephalus [13].

In patients with programmable valves, postoperative adjustment of the opening pressure may rely on the clinical findings rather than on the exact amount of intracranial air. In cases with symptomatic pneumocephalus temporary urgent treatment options include shunt ligation and air replacement via saline [17, 18]. If the formation of subdural hygromas is noted after resolution of pneumocephalus, elevation of the valve opening pressure, or in addition the implantation of an antisiphon device is an option.

In conclusion, our findings demonstrate that PFS in previously shunted patients is feasible at a relatively low perioperative risk.

## Data Availability

Data generated during or analysed during the current study, which are not included in this published article, are available from the corresponding author on reasonable request.

## Abbreviations

CSF: Cerebrospinal fluid
EVD: External ventricular drain
PFS: Posterior fossa surgery
SSP: Semi-sitting position
VA: Ventriculoatrial
VP: Ventriculoperitoneal
